# Increased incidence of seronegative autoimmune hepatitis in children during SARS-CoV-2 pandemia period

**DOI:** 10.3389/fimmu.2024.1445610

**Published:** 2024-09-11

**Authors:** Muriel Schmutz, Suzanne Chartier, Thierry Leblanc, Charlotte Mussini, Antoine Gardin, Emmanuel Gonzales, Anne-Marie Roque-Afonso, Solene Le Cam, Geraldine Hery, Benedicte Neven, Ramy Charbel, Jean-Pierre Vartanian, Emmanuel Jacquemin, Guillaume Morelle, Marion Almes

**Affiliations:** ^1^ Pediatric Hepatology and Pediatric Liver Transplant Department, Reference Center for Biliary Atresia and Genetic Cholestasis, FSMR FILFOIE, European Reference Networks (ERN) RARE LIVER, AP-HP, Paris-Saclay University, Bicêtre Hospital, Kremlin-Bicêtre, France; ^2^ Pathology Department, AP-HP, Paris-Saclay University, Bicêtre Hospital, Kremlin-Bicêtre, France; ^3^ Department of Pediatric Hematology and Immunology, AP-HP, Université Paris Cité Paris, Robert Debré Hospital, Paris, France; ^4^ INSERM Unité Mixte de Recherche (UMR)-S 1193, Paris-Saclay University, Hépatinov, Orsay, France; ^5^ European Reference Networks (ERN) Rare Liver, Hamburg, Germany; ^6^ Virology Department, National Reference Center for Hepatitis A virus, Paul Brousse Hospital, Assistance Publique-Hôpitaux de Paris (AP-HP), Villejuif, France; ^7^ Pediatric Radiology Department, AP-HP, Paris-Saclay University, Bicêtre Hospital, Kremlin-Bicêtre, France; ^8^ Department of Paediatric Surgery, AP-HP, Paris-Saclay University, Bicêtre Hospital, Kremlin-Bicêtre, France; ^9^ Pediatric Hematology-Immunology and Rheumatology Department, AP-HP, Université Paris Cité Paris, Necker-Children’s Hospital, Paris, France; ^10^ INSERM Unité Mixte de Recherche (UMR) 1163, Imagine Institute, Paris, France; ^11^ Pediatric Intensive Care Unit, AP-HP, Paris-Saclay University, Bicêtre Hospital, Kremlin-Bicêtre, France; ^12^ Virus and Cellular Stress Unit, Department of Virology, Institut Pasteur, Université de Paris Cité, Paris, France; ^13^ Centre for Haemophilia and Constitutional Bleeding Disorders, AP-HP, Paris-Saclay University, Bicêtre Hospital, Kremlin-Bicêtre, France; ^14^ Department of Pediatric Emergency, AP-HP, Paris-Saclay University, Bicêtre Hospital, Kremlin-Bicêtre, France

**Keywords:** pediatric seronegative autoimmune hepatitis, aplastic anemia, severe acute respiratory syndrome coronavirus 2, dysimmunity, immunosuppressive treatment

## Abstract

**Background:**

Seronegative autoimmune hepatitis in children is a rare but potentially severe disease, sometimes requiring liver transplantation. This type of hepatitis may be associated with various immunological and hematological disorders, ranging from isolated lymphopenia to aplastic anemia. Precise pathophysiological mechanisms are still unknown, but the role of viruses cannot be excluded, either as directly pathogenic or as triggers, responsible for an inappropriate immune stimulation. Having the impression of an increasing number of seronegative autoimmune hepatitis since the beginning of SARS-CoV-2 pandemia period, we hypothesized that SARS-CoV-2 virus could be an infectious trigger.

**Methods:**

We conducted a retrospective, observational, descriptive study about children with seronegative autoimmune hepatitis, in a tertiary care center, between 2010 and 2022.

**Results:**

Thirty-two patients were included. The overall incidence of seronegative autoimmune hepatitis increased 3.3-fold in 2020-2022, during the SARS-CoV-2 pandemia period (16 patients in 2.8 years) compared with 2010-2019 the pre pandemia period (16 patients in 9 years). Patients’ clinical and biochemical liver characteristics did not differ between the two periods. Hematological damages were less severe during the pandemia period. Immunological studies revealed a dysregulated immune response. The initiation of immunosuppressive therapy (corticosteroids ± cyclosporine) was earlier during the pandemia period than before.

**Conclusion:**

In cases of undetermined acute hepatitis, an immune-mediated origin should be considered, prompting a liver biopsy. If the histological aspect points to an immune origin, immunosuppressive treatment should be instituted even though autoimmune hepatitis antibodies are negative. Close hematological monitoring must be performed in all cases. The 3.3-fold increase of cases during the SARS-CoV-2 pandemia will need to be further analyzed to better understand the underlying immunological mechanisms, and to prove its potential involvement.

## 1 Introduction

Seronegative autoimmune hepatitis (SAIH) in children is a rare but potentially severe disease, mainly diagnosed through liver histology, after excluding the main causes of hepatitis (viral, drug-induced, autoimmune, metabolic). It has been reported to account for up to 3% of cases of hepatitis in children (1), and could likely be involved in 40 to 48% of cases of acute liver failure (ALF) (2–4). Hematologic abnormalities frequently follow the liver abnormalities. Previous studies have characterized these hepatitis (1, 5, 6), depending on IgG levels and the severity of the hematological features (inexistent, aplastic anemia (AA), peripheral thrombocytopenia with or without neutropenia) (1).

Associated hematological disorders could result from an immune mechanism combining sequential attack to liver and bone marrow (1, 5, 7). The role of viruses, either as directly pathogenic or as a trigger responsible for an inappropriate T-cell stimulation, cannot be excluded (8). AA, defined as the association of pancytopenia and bone-marrow hypocellularity without malignant, myeloproliferative, or fibrosis processes, may develop a few weeks after hepatitis (9). Hepatitis-associated AA was first described in 1955 (10). AA occurs in 30 to 33% of cases after liver transplantation (LT) for indeterminate ALF (4, 11) and in 0.07% of LT when indicated for acute viral hepatitis (12). The prognosis of the hematological complication is variable: some mild forms resolve spontaneously, while others require either immunosuppressive therapy, or hematopoietic stem cell transplantation (HSCT) (1, 13).

Having the impression of an increasing number of SAIH cases during the SARS-CoV-2 pandemia period, we hypothesized that SARS-CoV-2 virus could be a new infectious trigger for this disease (14, 15).

Our primary objective was to precise the clinical, biochemical and histological parameters of children with SAIH, and to report their overall outcome. Our secondary objectives were to compare the profiles of such children before and during the SARS-CoV-2 pandemia. Finally, we compared the children requiring LT and those who recovered without LT.

## 2 Materials and methods

### 2.1 Study design

We conducted an observational, retrospective, single-center study in the Pediatric Hepatology and Liver Transplantation Unit of a tertiary care center (CHU Bicêtre, France) between December 31, 2010, and September 30, 2022. The period “before SARS-CoV-2 pandemia” was defined by the 9 years prior to the pandemia, from December 31, 2010 to December 31, 2019. The period “during SARS-CoV-2 pandemia” was defined from January 2020, when the first case of SARS-CoV-2 virus infections were reported by Santé Publique France, to September 2022 (16).

### 2.2 Population

Inclusion criteria were: (a) age under 18 years old, (b) elevated serum aminotransferase activity, 5 times above the normal upper limit, with or without liver failure, (c) histological features compatible with SAIH as previously described (1).

Of note, 4 patients from the period “before SARS-CoV-2 pandemia” were already reported by Delehaye et al. [patients N°2, 3, 4,5 (17)].

Exclusion criteria were: (a) presence of specific autoantibody known to cause auto-immune hepatitis (anti-smooth muscle, antinuclear, anti-liver kidney-microsome 1, anti-liver cytosol 1) or celiac disease antibodies (anti-tissue transglutaminase IgA, total IgA) at a significant titer (≥ 1/160); (b) evidence of active viral infection by an hepatotropic virus (elevation of IgM antibody and/or blood PCR positivity, and/or PCR positivity on liver biopsy): hepatitis A virus, hepatitis B virus, hepatitis C virus, hepatitis E virus, Epstein-Barr virus, cytomegalovirus, adenovirus, enterovirus, herpes simplex virus type 1 and 2, human herpesvirus 6, parvovirus B19, human immunodeficiency virus; (c) drug induced hepatitis; (d) associated chronic liver disease. Date of diagnosis of hepatitis was defined as the day of liver biopsy.

We classified the associated hematological or immunological disorders into 4 categories: (a) AA defined as marrow cellularity below 30% on bone marrow biopsy (BMB) without evidence of AA causes (Fanconi anemia, telomeropathies and other inherited bone marrow failure syndromes, Paroxysmal nocturnal hemoglobinuria, viruses); (b) moderate cytopenia defined as bicytopenia i.e. decreased in 2 cell lines (hemoglobin < 9g/l and/or platelets < 150 000/mm3 and/or neutrophils < 1500/mm3) in the absence of bone marrow biopsy; (c) isolated lymphopenia without AA; (d) absence of hematological disorder. For the first two categories, the date of diagnosis of hematological disorder was the date of the bone marrow biopsy or aspiration (only one patient with moderate cytopenia did not have a bone marrow aspiration (BMA), the date of the most severe neutropenia was chosen) and for the third category, the date of onset of lymphopenia was chosen.

### 2.3 Data collection

Pediatricians with expertise in immunology and hepatology collected clinical and paraclinical data from patients’ charts.

Collected clinical items included: age, gender, autoimmune family history and consanguinity.

Regarding SARS-CoV-2 data, specific RT PCR results in nasopharyngeal secretions and antibodies (anti- nucleocapsid or spike protein) results were also collected, when available.

Collected liver parameters included: clinical data, serum alanine aminotransferase (ALT), aspartate aminotransferase (AST), gamma-glutamyl transpeptidase (GGT), total and direct bilirubin, serum bile acids, alpha-fetoprotein, prothrombin time (PT) and coagulation factor V (FV) levels. These data were reported at two different time points: on arrival, and when the PT value was at its lowest. Abdominal ultrasound and MRI (magnetic resonance imaging) findings, clinical and electrical signs (on electroencephalogram) of hepatic encephalopathy, maximum plasma ammonia rate and results of exhaustive viral screening data were also collected. When available, metagenomic next-generation sequencing performed on liver biopsy was collected.

Two different pathologists specialized in pediatric liver pathology performed a retrospective histological analysis of liver biopsies at diagnosis and, when available, of explanted native livers. They assessed inflammatory activity and fibrosis lesions for each case using the Ishak grading scheme (18) and METAVIR score, and biliary injury using the Kakuda score (19). Additional immunohistochemical staining of lymphocytic and plasma cell inflammatory infiltrate were performed on a Bond Leica automate, with anti-CD138 antibody (Agilent, clone MI15, dilution 1/100), anti-CD20 antibody (Agilent, clone L26, dilution 1/400), anti-CD3 antibody (Agilent, clone F7.2.38, dilution 1/50), anti-CD4 antibody (Leica, clone 4B12, pur), anti-CD8 antibody (Agilent, clone C8/144B, dilution 1/50).

A virologist analyzed each patient’ PCR (blood ± liver biopsy) and/or viral serology data.

The hematological and immunological items collected included, at the time of diagnosis of hepatitis: complete blood cells count, blood features of macrophage activation (fibrinogen, triglycerides, ferritin), serum levels of immunoglobulins, Coombs test, blood smear and blood lymphocyte immunophenotyping. We collected complete blood cells count at the lowest value of neutrophils (or lymphocytes if the patient didn’t have neutropenia). When performed, we reported the results of bone marrow aspiration and biopsy (cellularity, medullary invasion by malignant pathology, presence of hemophagocytosis and cell infiltration).

Treatment details and their indications were collected (steroids, cyclosporine, plasmapheresis, ursodeoxycholic acid, LT, anti-thymocyte globulin (ATG) and HSCT), as well as the time to normalization of serum liver tests and hematological parameters.

### 2.4 Statistical analysis

We performed a Poisson incidence rate ratio (IRR) analysis to compare the rates of seronegative autoimmune hepatitis before and during the pandemia with 95% confidence intervals. Comparisons of clinical and biological hepatic and hematologic features between the two groups “before pandemia” and “during pandemia” were performed using nonparametric tests (Wilcoxon test for quantitative variables, and Fisher test for qualitative variables). Data are exposed as median [IQR] or mean (SD). A comparison of clinical and biological liver characteristics of liver-transplanted (LTd) patients and non-LTd patients was also performed. All analyses were performed with Microsoft Excel and StatAid v1.2.4. A p value < 0.05 was considered significant. Missing data are specified in the tables.

### 2.5 Ethics

This study was carried out in accordance with MR004 reference methodology and complies with the General Data Protection Regulation (RGPD - Regulation (EU), No 20230630161435) and the French Data Protection Act. A non-opposition letter was sent to all parents to explain the purpose of the study, in accordance with the local guidelines established by the hospital’s ethics committee.

## 3 Results

### 3.1 Population characteristics

**Figure 1:**
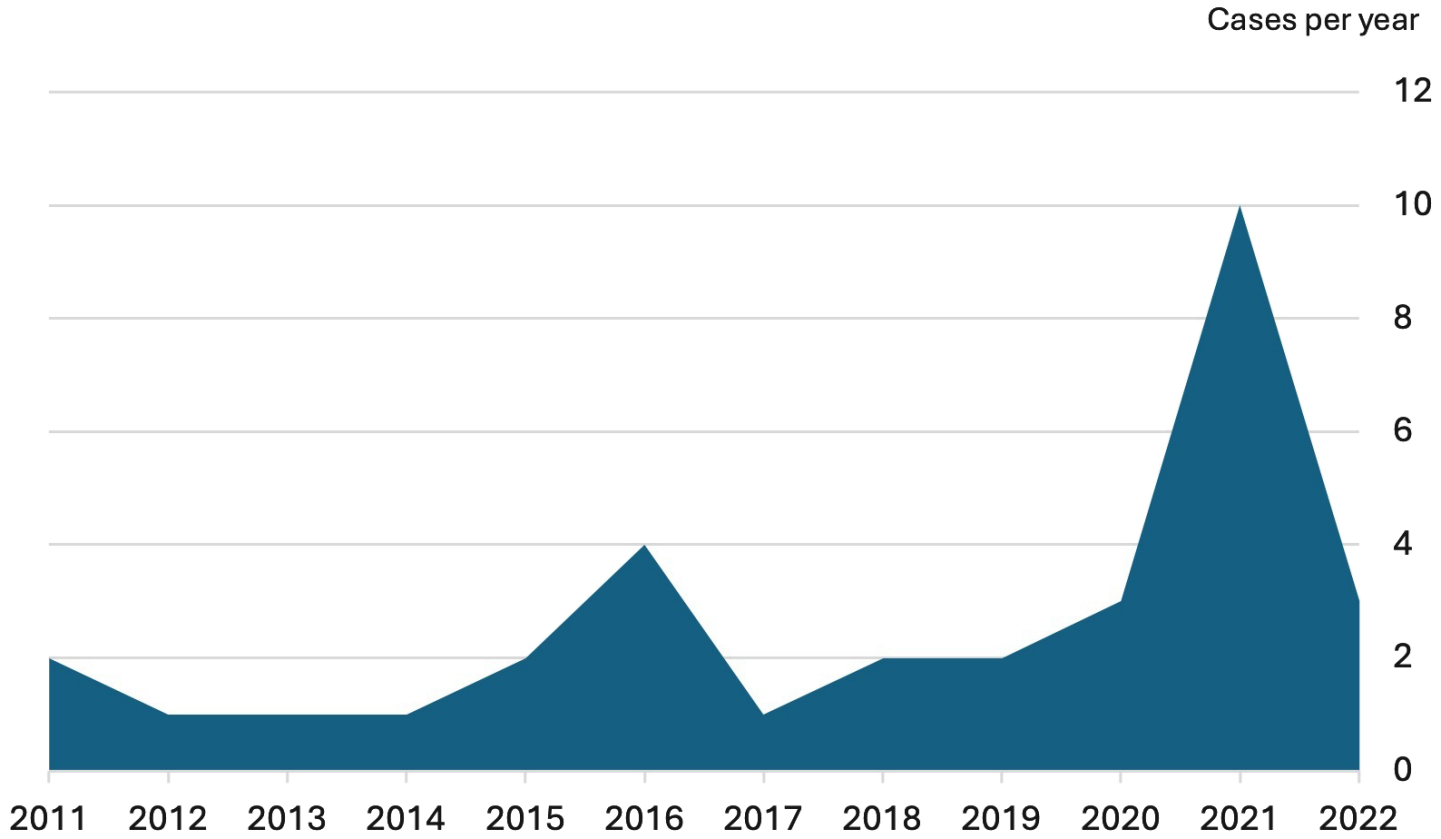
Incidence of seronegative autoimmune hepatitis.

Population characteristics are presented in **Table 1** and **Figure 1**. Thirty-five patients were identified between December 31, 2010, and September 30, 2022. Three patients were excluded because i) subsequent diagnosis of autoimmune hepatitis followed by idiopathic AA (n= 1), ii), diagnosis of hemophagocytic lymphohistiocytosis (n=1), and iii) of lacking data (n=1). The analyzed population ended in 32 patients.

**Table 1:** Patients characteristics.

	All patients 12/2010 - 09/2022 N = 32	Pre-pandemia period 12/2010-12/2019 N = 16	Pandemia period 01/2020-09/2022 N = 16
**Incidence of seronegative autoimmune hepatitis, n/year [CI]**	2.7 [1.86-3.84]	1.8* [1.01-2.89]	6* [3.40-9.69]
**Age at diagnosis, years, median, [IQR]**	7.5 [4.6-12.6]	7.1 [5.3-12.4]	7.9 [4.2-12.6]
**Sex ratio (M/F)**	2.3	2.4	2.2
**Familial history of autoimune disorder, n (%)**	9 (28)	4 (25)	5 (31)
**Sars-cov2 infection, n**	13	-	13
Positive Sars-cov2 RT PCR	0	-	0
Positive antibody	5	-	5
Nucleocapsids antibody	4	-	4
Spike protein antibody	5	-	5
**Associated hematological disease**			
Aplastic anemia, n (%)	16 (50)	11 (68)	5 (31)
Moderate cytopenia, n (%)	3 (9)	0	3 (18)
Isolated lymphopenia, n (%)	9 (28)	4 (25)	5 (31)
No hematological disease, n (%)	4 (12)	1 (6)	3 (18)
**Follow up years, median, [IQR]**	2 [1-5.2]	5.2 [3.7-7.1]	1.1 [0.9-1.4]

*p < 0.05, CI, 95% confidence intervals; IQR, interquartile range.

The incidence was of 1.8 cases per year (CI [1.01-2.89]) during the pre-pandemia period, and of 6 cases per year (CI [3.40-9.69]) during the SARS-CoV-2 pandemia period; representing a 3.27-fold increase (CI [1.64-6.54], p = 0.0008).

Sex ratio (male/female) and median age at diagnosis were similar for both periods (respectively 2.3 and 7.5 years). Nine children (28%) had a familial history of autoimmune diseases in first- or second-degree relatives (3 Graves’ disease, 2 vitiligo, 2 Crohn disease, 1 systemic lupus, 1 rheumatoid arthritis). No consanguinity was reported among the 24 patients with available data.

The median follow-up after diagnosis was 2 years (5.2 years for patients diagnosed before the pandemia period, and 1.1 year for patients diagnosed during the pandemia period).

Among the 16 patients diagnosed during the pandemia period, 13 samples were available for analysis SARS-CoV-2 antibodies: 5 of them (38%) presented SARS-CoV-2 spike (S) or nucleocapsid (N) IgG antibodies, and 8 were negative. Of note, none of our patients had been previously vaccinated against SARS-CoV-2. Nasopharyngeal RT PCR was performed in these 13 patients and were negative.

### 3.2 Liver characteristics

#### 3.2.1 – Clinical features and serum liver tests on arrival and at the time of lowest PT value

Clinical features and serum liver tests are in **Table 2A**. At admission, all patients had jaundice, 28 had hepatomegaly and only 4 had splenomegaly (without clinical signs of chronic liver disease or portal hypertension). They all presented high transaminase levels (AST median value: 1870 IU/L, IQR [1172-2417]), ALT median value: 1985 IU/L, IQR [1557-2299]) associated with a mild elevated GGT cholestasis (median value: 59 IU/L, IQR [45-86]). Total bilirubin, conjugated bilirubin and bile acids levels were elevated (median total bilirubin 305 µmol/L, IQR [230-366], median conjugated bilirubin 250 µmol/L, IQR [210-284]). Twenty-two patients (68%) presented with a PT level < 70% despite parenteral vitamin K supplementation. Five additional patients presented a drop in TP below 70% within few days. The median time from the arrival to lowest PT value was 4 days (IQR [0-6]).

**Table 2A:** Serum, liver tests, on arrival and at the time of lowest PT value.

	On arrival			At the time of lowest PT value		
	All patients 12/2010-09/2022 N = 32	Pre-pandemia period 12/2010-12/2019 N=16	Pandemia period 01/2020-09/2022 N=16	All patients 12/2010-09/2022 N = 32	Pre-pandemia period 12/2010-12/2019 N=16	Pandemia period 01/2020-09/2022 N=16
**Time to lowest PT value days, median, [IQR]**	–	–	–	4 [0-6]	4.5 [0.5-5.75]	3.5 [0-6.25]
**Liver biology, median, [IQR]**						
AST (IU/L)	1870 [1172-2417]	1852 [761-2189]	1933 [1470-3627]	1101 [593-2140]	1101 [375-1934]	1124 [639-2929]
ALT (IU/L)	1985 [1557-2299]	1985[1663-2435]	1985 [1663-2435]	1489 [851-2164]	1481 [798-1902]	1808 [951-2390]
GGT (IU/L)	59 [45-86]	60 [45-86]	59 [49-79]	46 [32-68]	45 [36-69]	46 [26-66]
Bilirubin						
Total (µmol/L) Conjugated (µmol/L)	305 [230-366] 250 [210-284]	357 [266-392] 270 [228-342.5]	293.5 [221-313] 247 [201-257]	326.5 [266-407] 253 [231-325]	390* [328-462] 283* [251-359]	285* [212-333] 236* [181-260]
Bile acids (µmol/L)	299 [265-426]	293 [240-360]	300 [271-427]	NA	NA	NA
PT < 70% (n) Range	22 9-69	9 9-69	13 23-69	27 8-69	13 8-64	14 10-69
PT < 20% (n) Range	3 9-18	3 9-18	0 .	8 8-17	5 8-17	3 10-11
FV < 80% (n) Range	9 16-77	4 16-73	5 38-77	15 3-74	8 3-73	7 10-74
FV < 20% (n) Range	1 16	1 16	0	4 3-15	2 3-11	2 10-15
**Other liver parameters**						
Encephalopathy, n	NA	NA	NA	12	5	7
Electrical grade, mean	NA	NA	NA	2,1	2	2,2
Ammonia max (µmol/L), median [IQR]	NA	NA	NA	157 [104-187]	166 [120-180]	136 [104-179]

*p < 0.05, ALT, Alanine aminotransferase; AST, Aspartate aminotransferase; GGT, gamma-glutamyltranspeptidase; IQR, interquartile range; LTd, liver transplanted; NA, Not available; Normal level of liver tests: AST and ALT < 45 IU/L, GGT < 25 UI/L, total bilirubin < 17 µmol/L, conjugated bilirubin < 10 µmol/L, ammonia < 50 µmol/L.

#### 3.2.2 Comparison of liver parameters in liver transplanted patients and non- liver transplanted patients

Comparison of liver parameters in liver-transplanted patients and non-liver-transplanted patients are presented in **Table 2B** and **Supplementary Table S1**. At admission, non-LTd patients presented significantly higher AST levels, PT and FV values (respectively p = 0.02, p < 0.001, p < 0.001) and lower alpha-foetoprotein levels (p= 0.01) than LTd patients.

**Table 2B:** Serum liver tests values of LTd patients and non-LTd patients.

	On arrival		At the time of lowest PT value	
	Non-LTd patients N = 24	LTd patients N = 8	Non-LTd patients N = 24	LTd patients N = 8
**Time to lowest PT value days, median, [IQR]**	–	–	4 [0-6.5]	4 [2-8.75]
**Liver biology, median, [IQR]**				
AST (IU/L)	2080* [1637-2779]	1194* [668-1817]	1476* [984-2204]	463* [281-682]
ALT (IU/L)	2059 [1575-2435]	1711 [1008-2037]	1926*[1440-2292]	790* [630-1077]
GGT (IU/L)	60 [46-86]	55.5 [45-79]	60* [41-74]	33* [26-37]
Bilirubin				
**Liver biology, median, [IQR]**				
Total (µmol/L) Conjugated (µmol/L)	299 [230-346] 250 [210-282]	335 [288-388] 257 [216-299]	311 [266-360] 250 [231-305]	388 [314-410] 289 [213-327]
Bile acids (µmol/L)	302 [271-426]	266.5 [234-359]	NA	NA
Alpha-fetoprotein (µg/L)	12* [6-25]	92* [58-92]	NA	NA
**Encephalopathy, n**	NA	NA	4	8
Electrical grade, mean	NA	NA	1,7	2,2
Ammonia max (µmol/L), median [IQR]	NA	NA	60* [44-84]	110* [95-135]

*p < 0.05, ALT, Alanine aminotransferase; AST, Aspartate aminotransferase; GGT, gamma-glutamyltranspeptidase; IQR, interquartile range; LTd, liver transplanted; NA, Not available; Normal level of liver tests: AST and ALT < 45 IU/L, GGT < 25 UI/L, total bilirubin < 17 µmol/L, conjugated bilirubin < 10 µmol/L, ammonia < 50 µmol/L.

At time of lowest PT value, non-LTd patients also presented significantly higher AST, ALT, GGT levels (respectively p = 0.001, p = 0.002, p = 0.007) and coagulation factors values (details S1). They had a lower ammonia level (P=0.02) than LTd patients. While 4 patients (16%) in the non-LTd group showed clinical (and/or electrical) signs of encephalopathy, 100% of patients in the LTd group had encephalopathy.

#### 3.2.3 – Etiological investigations

Etiological investigations conducted are presented in **Supplementary Table S** **2**. None of the patient showed any pattern of chronic liver disease on liver ultrasound. No evidence of chronic liver disease or biliary tract involvement was found on the seven MRI performed. None of the patients had significant auto-immune hepatitis specific antibodies. They had a median IgG level of 8 g/dl (**Supplementary Table S** **4**). The viral exhaustive investigation did not reveal any significative viral replication. Viral screening looked for hepatitis viruses (virus A-B-C-E) and for occasionally other hepatotropic viruses (Epstein-Barr virus, cytomegalovirus, adenovirus, enterovirus, herpes simplex virus type 1 and 2, human herpesvirus 6, parvovirus B19). Metagenomic next-generation sequencing performed on 2 liver biopsy samples did not detect any pathogen (no detection of bacteria, viruses, fungi or parasites) for patient n° 22 and n°26.

**Figure 2:**
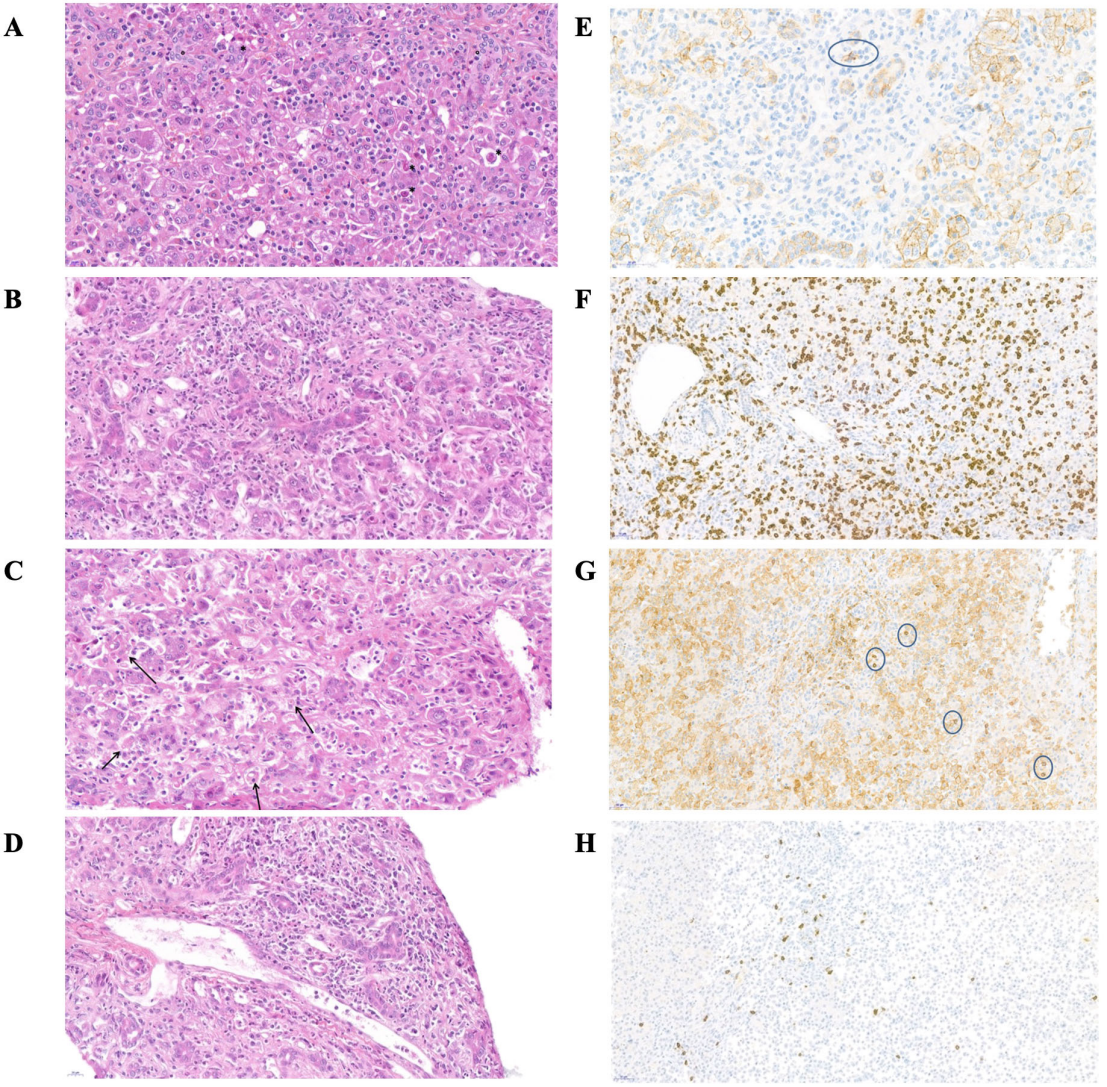
Histological features of the liver in patient n°17 at the time of initial presentation. **(A–D)** magnification of liver biopsy stained with hematoxylin and eosin. **(E–H)** immunohistochemical staining **(A)** Cell loss with acidophilic body (*), inflammation and ductular regeneration reaction (°). **(B)** Portal inflammation, diffuse T-lymphocyte and neutrophils infiltration **(C)** Hemophagocytosis **(D)** Portal endothelitis **(E)** Immunohistochemical stain for CD138+ (surrounded plasma cell, no significant) **(F)** Immunohistochemical stain for CD8 **(G)** Immunohistochemical stain for CD4 (surrounded lymphocytes) **(H)** Immunohistochemical stain for CD20.

Twenty-eight liver biopsies and 4 liver explants were examined (**Figure 2**; **Supplementary Table S5**). In all cases, inflammation assessment using Ishak score demonstrated a high degree of necrotic inflammatory activity: at least moderate to marked interface activity (≥ A3) in 26 patients, confluent necrosis in zone 3 (pericentrolobular) or beyond (≥ B3) in 28 patients, and more than 5 lobular foci with activity (≥ C4) in 27 patients. Twenty-six biopsies showed severe portal inflammation with a score of D3 or D4. METAVIR score was also in favor of an inflammatory aggressiveness in all biopsies (METAVIR A2 = 3 patients; A3 = 27 patients). Fibrosis was reported at a variable extent in 28 biopsies (METAVIR F1/2 = 20 patients, METAVIR F3 = 8 patients). The Kakuda score revealed mild to moderate biliary cholangitis, without evidence of sclerosing cholangitis in most cases (bile duct loss in less than one-third of portal tracts in 3 biopsies; 1 bile duct with evident chronic cholangitis in 8 biopsies; and more than 2 bile ducts with evident chronic cholangitis in 18 biopsies).

In most patients, common features of the inflammatory infiltrate were: diffuse T-CD8+ lymphocyte infiltration in the portal area and lobules; rare CD20+ B-lymphocytes, mostly in the portal area and without tertiary lymphoid organization, numerous neutrophils. No significant plasma cell component was observed on light microscopy (immunostaining of CD138+ plasma cells revealed a moderate infiltrate in 2 patients). All but three biopsies showed signs of central venous and/or portal endothelitis, with no evidence of constituted hepatic veino-occlusive disease (no central venous obstruction or centrilobular sinusoidal dilatation). A ductular reaction of variable intensity was present on all biopsies, associated with cholestasis (canalicular plugs), and rosettes or pseudogland. All patients had similar biopsies, regardless of the inclusion period (before or during pandemia) or the presence of hematological complications. For all children with a liver biopsy before LT, a comparison between their biopsy and their explanted liver was carried out (after 2-15 days of corticosteroid ± 2-4 days of cyclosporine therapy): no difference was found, apart from a decrease of the neutrophil infiltrate. Of note, 23 patients showed signs of hemophagocytosis on their liver biopsies at diagnosis.

### 3.3 Immuno-hematological characteristics

At the time of diagnosis of hepatitis, median value of hemoglobin, platelet and leucocyte were normal, but 6 patients already presented at least one cytopenia (anemia, thrombopenia or neutropenia) (**Table 3**). Lymphopenia was noted in 21 of 32 patients (65%). No difference in lymphocyte count was found between groups with and without AA. At the time of the lowest value (lowest neutrophil and/or lymphocyte count), patients had a marked decrease in the median platelet count (40 G/L, IQR [13-246] vs 249 G/L, IQR [201-312]) (**Table 3**).

**Table 3:** Hematological characterization.

	At the time of diagnosis of hepatitis			At lowest value (neutrophils or lymphocytes)		
	All patients 12/2010-09/2022 N = 32	Pre-pandemia period 12/2010-12/2019 N=16	Pandemia period 01/2020-09/2022 N=16	All patients 12/2010-09/2022 N = 32	Pre-pandemia period 12/2010-12/2019 N=16	Pandemia period 01/2020-09/2022 N=16
**Time to nadir, days, median, [IQR]**	-	-	-	25 [18-75]	67 [17-97]	25 [20-56]
**Blood count, median, [IQR]**						
Hb (g/dL)	11 [10-12]	12 [10-12]	11 [10-12]	10 [8-11]	10 [8-11]	9 [8-11]
MCV (fL)	78 [75-81]	78 [74-81]	78 [76-80]	82 [79-83]	83 [81-84]	79 [76-82]
Reticulocytes (G/L)	87 [71-135]	85 [68-135]	102 [71-128]	45 [26-83]	23* [10-44]	69* [44-89]
WBC (cells/mm3)	5720 [4640-6777]	5450* [3800-6040]	6385* [5285-7547]	1845 [1072-5025]	1075* [952-2467]	3335* [1837-5867]
PMN (cells/mm3)	3300 [2377-4030]	3130 [2085-3723]	3400 [2717-4217]	935 [500-3715]	760 [355-1080]	1190 [710-4060]
Lymphocytes (cells/mm3)	1260 [757-1940]	795* [502-1090]	1765* [1270-2967]	700 [340-825]	710 [265-870]	680 [385-822]
Platelets (G/L)	249 [201-312]	248 [66-291]	251 [218-324]	40 [13-246]	17* [12-55]	239* [25-303]

*p < 0.05, Hb, hemoglobin; IQR, interquartile range; PMN, neutrophilic polynuclear; MCV, Mean corpuscular volume; WBC, white blood cells.

None of the patients had criteria for biological macrophage activation syndrome at the time of diagnosis of hepatitis. Blood smears did not show any abnormal cells or blasts.

Twenty-one bone marrow aspirations (BMA) were performed within a median of 45 days from the diagnosis of hepatitis (IQR [12-70], **Supplementary Table S3**). The BMA suggested AA in 12 patients with low cellularity. Among 7 patients with normal cellularity on BMA, 3 patients were finally diagnosed AA on bone marrow biopsy (BMB). Two patients with moderate cellularity on BMA also had AA on BMB. All the BMB performed in the 16 patients confirmed the diagnostic of AA and eleven of them showed an inflammatory infiltrate composed of CD8+ T lymphocytes.

Overall, 16/32 patients developed AA (50%), 3 developed moderate cytopenia without diagnostic of AA, 9 presented an isolated lymphopenia, and 4 did not present any immunologic or hematologic disorder. Of note, one patient with poor cellularity on BMA was not diagnosed as AA due to the absence of a BMB and was classified as moderate cytopenia. In 87% (14/16 patients) of AA cases, liver abnormalities preceded hematological disorder. Two patients (n°10 and n°16) presented a hematological disorder respectively 3 and 6 days before hepatitis (**Tables 4A**, **B**).

**Table 4A:** Patient management and outcome – pre-pandemia period (N = 16).

Outcomes and management of liver disease							Outcomes and management of hematologic disease				
Patients		Corticosteroid			Other treatment of hepatitis	Days from treatment of hepatitis to normalization of liver tests	Haematological disorder	Days from hepatitis to hematological disorder	Additional treatment of hematological disease	Days from hematological disease to treatment	Months from hematological treatment to normalization of blood count cell
		Days from arrival to treatment	Indication	Dose (mg/kg/d)							
LTd	1	6	Hepatitis	NA	LT Day 18 AUDC	140	Isolated lymphopenia	0	Not interpretable ***^a^***		9,8
	2	33	None before LT - Graft rejection	1	LT Day 2 AUDC	321	AA	23	ATG-CSA	4	2,2
	3	25	None before LT - Graft rejection	5	LT Day 2 AUDC	NA	AA	16	HSCT	60	8,9
	4	78	None before LT - Graft rejection	5	LT Day 1 AUDC	314	AA	183	ATG-CSA	33	20
	5	7	Hepatitis	2	LT Day 23 AUDC	NA	AA	62	ATG-CSA	28	33,5
No LTd	6	–	–	–	AUDC	234 ***^b^***	AA	121	ATG-CSA	8	7,7
	7	9	Hepatitis	1	AUDC	71	AA	74	HSCT	99	1,7
	8	–	–	–	–	80 ***^b^***	None	–	–	–	–
	9	6	Hepatitis	1,2	–	63	AA	65	ATG-CSA	8	2,8
	10	7	MAS	2	AUDC	199	AA	-3	CSA	16	3,8
	11	1	Hepatitis	2	AUDC	93	AA	81	ATG-CSA	2	3,1
	12	9	Hepatitis	1	AUDC	118	Isolated lymphopenia	0	–	–	15,8
	13	8	Hepatitis	2	AUDC	54	AA	48	ATG-CSA	43	NA
	14	11	Hepatitis	1	–	40	Isolated lymphopenia	2	–	–	7,4
	15	5	Hepatitis	2	CSA	112	Isolated lymphopenia	0	–	–	8,9
	16	302	Hepatitis	0,6	AUDC	NA	AA	-6	CSA	11	10,1

AA, Aplastic anemia; ATG, Antilymphocyte Globulin; AUDC, ursodeoxycholic acid; CSA, Cyclosporine; CTC, corticosteroid; HSCT: hematopoietic stem cell transplantation; MMF, mycophenolate mofetil NA, not available; LFU: last follow up; LT, liver transplantation; LTd, liver transplanted; MAS, macrophagic activation syndrome; ^a^ Not interpretable: For this patients, anti-rejection immunosuppressive treatments were initiated after the liver transplantation. Hematological abnormalities appeared under tacrolimus. ^b^ normalization without treatment.

**Table 4B:** Patient management and outcome – pandemia period (N = 16).

Outcomes and management of liver disease							Outcomes and management of hematologic disease					
Patients		Corticosteroid			Other treatment of hepatitis	Days from treatment of hepatitis to normalization of liver test	Haematological disorder	Days from hepatitis to hematological disorder	Additional treatment of hematological disease	Days from hematological disease to treatment		Months from hematological treatment to normalization of blood count cell
		Days from arrival to treatment	Indication	Dose (mg/kg/d)								
LTd	17	2	Hepatitis	2	LT Day 4, AUDC	33	Isolated lymphopenia	0	Not interpretable ***^a^***			Abnormal at last FU
	18	2	Hepatitis	2	LT Day 7, CSA Plasmapheresis,	Anormal at last consultation	Moderate cytopenia	55	Not interpretable ***^a^***			4,9
	19	1	Hepatitis	2	LT Day 7, CSA Plasmapheresis	194	Isolated lymphopenia	5	Not interpretable ***^a^***			0,8
No LTd	20	7	Hepatitis	2	Azathioprine	Anormal at LFU	Isolated lymphopenia	0	–	–		Abnormal at last FU
	21	4	Hepatitis	2	AUDC	52	None	–	–	–		–
	22	6	Hepatitis	2	AUDC	NA	AA	45	MMF	-10		NA
	23	8	Hepatitis	2	AUDC	42	Isolated lymphopenia	0	–	–		0
	24	2	Hepatitis	1	–	74	AA	74	ATG-CSA	10		8
	25	6	Hepatitis	2	AUDC	35	None	–	–	–		–
	26	4	Hepatitis	1,5	AUDC	110^c^	Isolated lymphopenia	1	–	–		0,1
	27	5	Hepatitis	2	AUDC	83	Moderate cytopenia	22	CSA	9		0,7
	28	3	Hepatitis	1	AUDC, CSA, Plasmapheresis	102	Moderate cytopenia	28	–	–		6,4
	29	6	Hepatitis	2	AUDC	62	AA	113	ATG-CSA HSCT n°1 HSCT n°2	26 183 288		NA
	30	8	Hepatitis	1	AUDC	78	AA	26	HSCT	51		5,7
	31	–	–	–	AUDC	127 ***^b^***	AA	15	HSCT	46		2,1
	32	–	–	–	–	177 ***^b^***	None	–	–	–		–

AA, Aplastic anemia; ATG, Antilymphocyte Globulin; AUDC, ursodeoxycholic acid; CSA, Cyclosporine; CTC, corticosteroid; HSCT, hematopoietic stem cell transplantation; MMF, mycophenolate mofetil; NA, not available; FU: follow up; LT, liver transplantation; LTd, liver transplanted; MAS, macrophagic activation syndrome ^a^ Not interpretable: For these 3 patients, anti-rejection immunosuppressive treatments were initiated after the liver transplantation. Hematological abnormalities appeared under tacrolimus, cellcept or cyclosporin. ^b^ normalization without treatment. ^c^ Recurrence of hepatitis at one year of diagnosis associated with isolated tempory lymphopenia, improved by increasing corticosteroid doses and introducing cyclosporin.

Twenty-seven patients underwent a standard lymphocyte immunophenotyping, which showed generalized lymphopenia (low CD3+ cells), a decrease in CD4+ and an almost normal median CD8+ rate (**Supplementary Table S4**). Six of them underwent a complete immunophenotyping, which revealed an increase in CD45RA- CCR7- effector memory CD8+ at the expense of CD45RA+CCR7+ naïves CD8+ T cells. Immunoglobin levels was normal with a median IgG level of 8 g/L (IQR [6.7-11.1]).

### 3.4 Patient management and outcome

Patient management and outcome are presented in **Tables 4A**, **4B**. The first line of immunosuppressive treatment for hepatitis was corticosteroids alone in 21 patients, corticosteroids in combination with cyclosporine in 2 patients, and corticosteroids in combination with azathioprine in 1 patient. Twenty-two patients received ursodeoxycholic acid, 2 of them as monotherapy for their hepatitis. In 2 patients, liver function improved spontaneously without any treatment. Finally, 3 patients underwent LT directly and 4 others after a short course of corticosteroids. The median time between arrival and LT was 5,5 days. Return to normal liver test for non-LTd patients was obtained in 80 days (median, IQR [62-112]).

The 16 patients with AA received specific treatment within a median time of 26 days after diagnosis of AA. Nine were treated with anti-thymocyte globulin (ATG) and cyclosporine; 2 with cyclosporine alone, 1 with mycophenolate mofetil. Four patients required allogenic HSCT and received bone marrow from HLA- matched related donor. The hematological response to immunosuppressive therapy was defined as in international guidelines (20). After 3 months of treatment, 3 patients were in complete remission (CR) and 5 patients in partial remission (PR), 2 non-responders and 2 had missing data. After 6 months, 5 patients were in CR, 3 in PR and 2 non-responders and still 2 had missing data. With a median follow up of 2 years no difference in global outcome was noted between patients treated medically and those who underwent HSCT. It should be noted that patient n°3 received a liver transplant and then an allogenic HSCT 2 and a half months later. She is now well, 7 years post-transplants.

Three patients were diagnosed moderate cytopenia while they were treated with immunosuppressive treatment for their hepatitis or for antirejection drugs. Two of them improved after intensification of their immunosuppressive treatment (patient n° 18 with corticosteroid and patient n° 27 with cyclosporine). The third continued treatment with corticosteroids and cyclosporine, which were indicated for his hepatitis, and his complete blood count improved in 6.4 months.

After a median follow-up of 2 years, all patients were well and alive.

Of note, patient n° 26 presented a relapse of SAIH (proven on liver histology and serum liver tests) one year after the initial hepatitis, while he was in a decreasing phase of steroid therapy. Interestingly, he had undergone a SARS-CoV-2 infection (positive nasopharyngeal PCR and SARS-CoV-2 spike (S) and nucleocapsid (N) IgG antibodies) about 2 months before. This relapse was associated with a transient and isolated lymphopenia. Both immunological and hepatological disorders normalized within 35 days, after an increase in steroid doses and introduction of cyclosporine.

### 3.5 Comparison of hepatological, hematological and immunologic features of the patients before and during the pandemia period

While hepatological features of the patients were similar between the two periods, cytopenia were deeper before the pandemia period (**Tables 2A**, **3**; **Supplementary Table S5**). At the time of the lowest value (lowest neutrophil and/or lymphocyte count), platelet, leucocyte and reticulocyte counts were statistically lower before the pandemia period than during the pandemia (respectively, p = 0.018, p = 0.025, p = 0.03). On the time of diagnosis of hepatitis, lymphopenia was also significantly more pronounced before the pandemia period (p = 0.001) (**Supplementary Table S4**).

Regarding the management of patient, an earlier introduction of corticosteroids was noted during the pandemia period, within a median time of 4.5 days (IQR [2.2-25.6]) vs 8.5 days (IQR [6.2-21.6]) from onset of hepatitis (p = 0.004) (**Tables 4A**, **B**). Fewer patients required a liver transplantation during the pandemia period (3 patients vs 5 patients).

## 4 Discussion

This retrospective study describes 32 children with SAIH diagnosed in a pediatric hepatology unit between December 31, 2010, and September 30, 2022. As previously reported in literature, our cohort included a majority of boys with an median age of 7.5 years old (2, 3).

To the best of our knowledge, there is no data in the literature identifying a causal agent explaining the development of SAIH. In particular, no association with any hepatotropic viruses has been reported (7, 21). The hypothesis of parvovirus B19 involvement has often been raised, since it is known to be involved in both liver and bone marrow disorders (22). This virus was detected in 3 patients of this cohort, but at levels suggesting persistence of viral DNA in the tissues after an infection rather than an active infection, as previously described (23). Despite recent studies reporting potential implication of Adeno-associated virus 2 (AAV-2), it was not detected in any patient of this study (24, 25).

Regarding liver damage, patients’ biological and histological features did not differ between both periods except for bilirubin levels, which were higher before SARS-CoV-2 pandemia. This point was already reported by Leiskau et al. (40). Precise analysis of histological features has allowed us to better characterize SAIH, compared to seropositive autoimmune hepatitis. First, inflammatory infiltrate is different and do not contain a significant plasma cell contingent (1, 18). Moreover, when signs of activity are mainly located in periportal and centro-lobular areas in seropositive autoimmune hepatitis, here they are noted in periportal, central and medio-lobular compartments. Finally, many neutrophils, as well as central and portal endothelitis were noted, which are not usual aspects of seropositive autoimmune hepatitis. Of note, 8 patients had a METAVIR score of F3, but this score probably overestimates the degree of fibrosis due to the significant hepatocellular loss in these patients. Histological analysis of liver biopsies is therefore an essential part of the diagnosis in SAIH, and should be performed in all patient with indeterminate acute hepatitis, whether they present ALF or not (41, 42). Identified poor outcome parameters (lower transaminases and GGT levels and elevated AFP at diagnosis) were concordant to those reported in other series (28, 40).

In this study, all patients had normal IgG levels but exhibited various hematological complications (50% of them developing AA, 9% only moderate cytopenia); as reported by Maggiore et al. (group 3 and 4) (1). AA appeared in a median time of 26 days after the onset of hepatitis, as previously described (3, 9, 13). In contrast to other studies, lymphopenia was not identified as a predictive factor for AA in patients with SAIH (1, 17). Hematological disorders were less severe during the pandemia period, during which patients mostly presented isolated lymphopenia or moderate cytopenia. These less severe hematological disorders in patients from the pandemia period may be attributed to earlier and more intensive immunosuppressive therapies since 2020. For instance, 3 patients with ALF were treated with plasmapheresis and cyclosporine, which is known to be the most effective drug in AA (17)). However, further studies are needed as previous studies reported that immunosuppressive therapies (tacrolimus, cyclosporine or mycophenolate mofetil) were insufficient to prevent AA in children with indeterminate ALF (17). Other pathophysiological mechanisms leading to less severe hematological damage cannot be excluded.

Although the treatment of indeterminate ALF remains mainly symptomatic, most teams treat those patients with corticosteroids. The first study reporting efficacy of corticosteroids (combined with intravenous immunoglobulins) in SAIH showed an initial biological improvement in the 9 patients studied and 5 of them recovered with their native liver (41). Another study reported 34 patients with indeterminate ALF or hepatitis-associated AA, and showed a decrease in mortality from 15% to 3.5%, but no change in LT rate, depending on whether they used corticosteroids or not (43). In this study, 75% of the patients received corticosteroids for SAIH, with an earlier introduction of this treatment over the last 2 years. Moreover, cyclosporine and plasmapheresis have likely enabled to avoid LT in one patient, despite severe ALF. Kelgeri et al. reported 44 patients with similar presentation (unconclusive exhaustive etiological investigations, CD8+ T cell infiltrate in liver biopsies). Despite short follow-up and missing data, 38 patients experienced spontaneous improvement (defined as resolving liver dysfunction), while 6 required LT (28). With a median follow-up of 2 years, all our patients are well and alive. To be noted, median follow-up of patients from pandemia period is shorter (median 1.1 [0.9-1.4] years) and deserves long term studies.

We noted a marked increase in the incidence of SAIH since 2020, raising the hypothesis that SARS-CoV-2 may be involved. The incidence of SAIH, estimated at 1.36 cases/year between 1988 and 2010 in the study of Maggiore et al. (1), is similar to what we found during the pre-pandemia period of our study, between December 31, 2010, and December 31, 2019, with 1.8 cases/year. It was enhanced by a 3,3-fold since January 2020, corresponding to the beginning of the SARS-CoV-2 pandemia. World Health Organization (WHO) and the European Centre for Disease Prevention and Control (ECDC) have raised alerts regarding hepatitis outbreaks, particularly among children, since October 2021 (14, 15). A review of the literature had identified 1643 cases of indeterminate hepatitis in children in Europe, USA, Israel and India from April 1^st^ 2021 to August 30^th^ 2022 (24). Among this large cohort of patients, the rate of positive PCR for SARS-CoV-2 varies between 7 and 28% (24, 26–28). The worldwide increase in cases of indeterminate hepatitis must be analyzed with caution as etiological investigations were not always complete, with a lack of biological data and especially rare liver histology. One strength of our study is the precise search for differential diagnoses and the systematic performance of liver biopsies. Examination of the biopsies by two specialized pathologists revealed that liver histological features were similar in both periods, and regardless of the associated hematological disorders.

We investigated immunological and microbiological data of all patients to try to establish a link between SARS-CoV-2 infection and the development of SAIH. Although none of our patients had been previously vaccinated against SARS-CoV-2, four exhibited both SARS-CoV-2 spike (S) and nucleocapsid (N) IgG antibodies, indicating a previous infection. One patient (n° 26) displayed only spike IgG antibodies, suggesting either a prior natural infection with subsequent loss of nucleocapsid antibodies, or a very recent infection. This last hypothesis is though very unlikely since his nasopharyngeal SARS-CoV2 PCR testing was negative. Interestingly, this same patient had a second SARS-CoV-2 infection, and about 2 months later, a relapse of SAIH associated with lymphopenia. He had a favorable outcome after an increase of his corticosteroid doses and introduction of cyclosporine. To our knowledge, only one similar case of relapse was previously reported, before pandemia period (44). Regarding the remaining 8 patients with negative serologies, a defect in B cells function could be suspected, but all patients had normal B cell counts, as well as serum immunoglobulin levels. The most probable explanation to these negative serologies is that routine serological tests at the beginning of the pandemia were lacking sensitivity, as demonstrated by Avramescu et al. in post-COVID acute tubulointerstitial nephritis (39).Unfortunately, we do not have blood samples from these patients at diagnosis to re-test SARS-CoV2 serologies with the current more efficient techniques. Moreover, we do not have any serologies for 3 patients without any possibility of testing them retrospectively. This is the main limitation of our study, due to its retrospective design.

Various mechanisms have been suggested for how viral infections, particularly SARS-CoV-2, might induce dysimmune and autoimmune conditions: dysregulation of the innate and adaptative immune response, exuberant inflammatory reactions, bystander activation, molecular mimicry and genetic predisposition (29, 30). Dysregulation of the innate immune system has been widely described as a consequence of SARS-CoV-2 infection (29). Innate immune responses and specifically the IFN type 1 pathway is known to be essential in controlling these viral infections as highlighted by severe SARS-CoV-2 lung disease due to deficiencies in innate immunity or neutralizing antibodies against IFN type 1 (31) Worldwide, the virus is known to have an immunomodulatory role in children, as evidenced by the emergence of Kawasaki-like multi-systemic inflammatory syndrome reflecting a cytokine storms following SARS-CoV-2 infection (32–34). Molecular mimicry, where T cells recognize and react to both viral antigens and self-antigens with structural or sequential homology, has also been reported as a mechanism of virus-induced immune disease (30). A recent study identified T cells with TCRs reactive to both common viruses (CMV, EBV) and self-antigens on hematopoietic progenitor cells, explaining T cell-mediated elimination of these cells in aplastic anemia (8). This could explain that our patients suffer from T cell–mediated liver and bone marrow injury. The immune phenotype of SARS-CoV-2 infected patients also highlights overactivation of natural killer cells and CD8+ T cells, along with dysregulation of B and T cells, potentially leading to autoimmune reactions (30, 35). SARS-CoV-2 vaccines have been linked to an immunomodulatory effect, with instances of autoimmune hepatitis and even with intrahepatic accumulation of activated CD8 T cells with SARS-CoV-2 specificity reported in one case (36–38).

Our study highlights that immuno-mediated origin must be suspected and investigated in pediatric patients presenting an acute hepatitis, even when they do not present the classic autoimmune hepatitis autoantibodies. A liver biopsy should thus be performed as soon as noninvasive investigations (autoantibodies and main hepatotropic viruses PCR) show negative results. In addition to immunosuppressive treatment, a close follow-up of these patients is required to detect potential hematological complications and to intensify immunosuppressive treatments when confirmed. The role of SARS-CoV-2 as a possible trigger of these SAIH is suspected because of the increase of their incidence during the pandemia, but remains to be confirmed through further studies, especially characterizing the immunological mechanisms involved.

## Data Availability

The datasets presented in this study can be found in online repositories. The names of the repository/repositories and accession number(s) can be found in the article/**Supplementary Material**.
