# Supplementary material for: Increased incidence of seronegative autoimmune hepatitis in children during SARS-CoV-2 pandemia period
Source: Front Immunol. 2024 Sep 11;15:1445610. doi: 10.3389/fimmu.2024.1445610 (PMC11425678; doi:10.3389/fimmu.2024.1445610)
Supplement: Supplementary file 1 [file DataSheet1.docx]

Supplementary Material

Increased incidence of seronegative autoimmune hepatitis in children during SARS-CoV-2 pandemia period

Schmutz M.^1*^, Chartier S.^2^, Leblanc T^3^., Mussini C. ^2^, Gardin A. ^1, 4, 5^, Gonzales E.^1, 4, 5^, Roque Afonso A-M.^4,6^, Le Cam S.^7^, G Hery^8^, Neven B^9,10^, Charbel R^11^, Vartanian J-P.^12^, Jacquemin E.^1, 4, 5^, Morelle G.^13,14^, Almes M.^1, 4, 5^

*** Correspondence:** Muriel Schmutz, muriel.schmutz@aphp.fr

**S1- Comparison of hemostasis factors of future liver-transplanted (LTd) patients and non-LTd patients, all periods combined.**

| **Hemostasis factor on arrival** | | **Hemostasis factor at the lowest PT value** | |
| --- | --- | --- | --- |
| 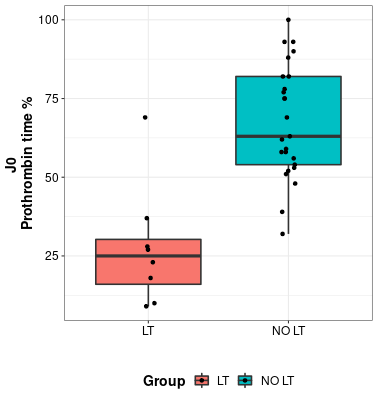 | 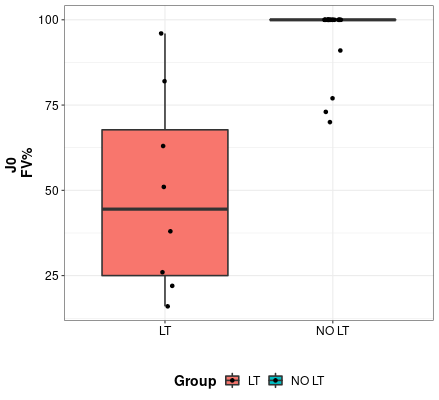 | 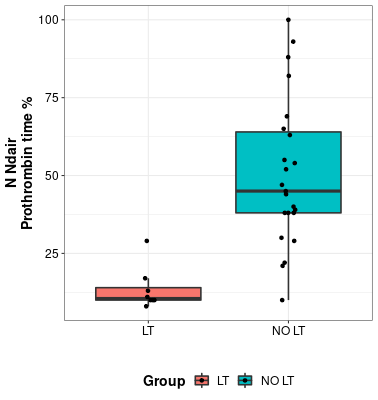 | 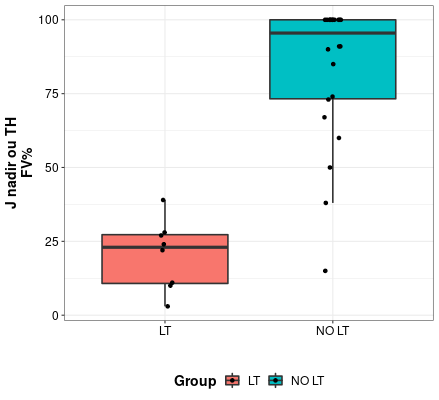 |
| P < 0.001 | P < 0.001 | P < 0.001 | P < 0.001 |

LT : liver transplantation, LTd : liver transplanted patient, FV : coagulation factor V

**S2 - Results of etiological investigations in the 32 patients with a seronegative autoimmune hepatitis**

| **Test** | | **Total (32 patients)** |
| --- | --- | --- |
| Drug Toxicity: history and/or serum acetaminophen level measured | | 0 /32 |
| Screening for Autoimmune Hepatitis | Antinuclear antibody | 2 /32 (titler: 1/160 - without specificity) |
|  | Anti-smooth muscle antibody | 0 / 32 |
|  | Anti-liver kidney-microsome 1 antibody | 0 / 32 |
|  | Anti-liver cytosol 1 antibody | 0 / 32 |
|  | Anti-mitochondrion 1 antibody | 0 / 32 |
| Screening for Infections | HBV (HBs Ag and anti HBc) | 0 / 32 |
|  | Anti-hepatitis A / C virus IgM | 0 / 32 |
|  | Anti-hepatitis E IgM and/or blood PCR | 0 / 32 |
|  | EBV anti VCA IgM and/or blood PCR | 3 / 32 (2 patients with suspicions of primary EBV infection (IgM VCA+ and PCR 3log and 4log for each patient), 1 patient with PCR 2,8log and negative serology) |
|  | CMV anti IgM and/or blood PCR | 0 / 32 |
|  | HSV blood PCR | 1 / 26 (PCR 4log) |
|  | HHV6A/B blood PCR | 8 / 31 (all patients with weakly positive PCR < 3log) |
|  | HHV8 blood PCR | 0 / 29 |
|  | Enterovirus blood PCR | 1 / 30 (patient with weakly positive PCR < 3log) |
|  | Adenovirus blood PCR | 1 / 29 (patient with weakly positive PCR < 3log) |
|  | Parvovirus B19 blood PCR | 3 / 27 (all patients with weakly positive PCR < 3log) |
|  | VIH infection | 0 / 24 |
|  | PCR on liver biopsy | 16 patients with available data  1/16 patient with HHV6B replication on liver biopsy  1/16 patient with parvovirus B19 replication on liver biopsy |
| Underlying chronic liver disease: history and ultrasound information  (including Wilson’s disease, alpha-1-antitrypsin deficiency and other metabolic disease) | | 0 |

VHB: hepatitis B virus EBV: Epstein Barr virus, CMV: cytomegalovirus, HSV : herpes simplex virus, HHV 6 and 8 : human herpesvirus type 6 and 8, VIH : human immunodeficiency virus

**S3 - Bone marrow aspiration and biopsy**

|  | **All patients**  12/2010-09/2022  N = 32 |
| --- | --- |
| **Bone marrow aspiration (BMA), n (%)** | 21 (65%) |
| Time from diagnosis of hepatitis to BM aspiration (days, median [IQR]) | 45 [12-70] |
| Description of cellularity  Normal  Moderate  Poor | 7 (21%)  2 (6%)  12 (37%) |
| Blasts | 0 |
| Hemophagocytosis | 4 (12%) |
| **Bone marrow biopsy (BMB), n (%)** | 16 (50%) |
| Time from diagnosis of hepatitis to BMB days, median [IQR]) | 60 [23-78] |
| Aplastic anemia (cellularity < 30%) | 16 (50%) |
| Myelofibrosis | 1 (3%)^1^ |
| Lymphatic infiltrate | 11 (34%) |

BMA: bone marrow aspiration, BMB: bone marrow biopsy

^1^ one patient had systematized myelofibrosis on 10% of the surface

**S4 – Blood lymphocyte immunophenotyping and serum immunoglobulin levels**

|  | **All patients**  12/2010-09/2022 | **Pre-pandemic period**  12/2010-12/2019 | **Pandemic period**  01/2020-09/2022 | Standard for children 6 to 12 years old |
| --- | --- | --- | --- | --- |
| **Blood lymphocyte phenotyping, n** | 27 | 14 | 13 |  |
| Total lymphocyte, median [IQR] | 1016 [701-1504] | **800*** [623-1011] | **1242***[1035-2047] | 1900-3700 |
| **CD3^+^ (cells/ μl)** | 532 [391-1271] | 455 [374-599] | 770 [507-2413] | 1200 - 2600 |
| **CD4^+^ (cells/ μl)**  CD31^+^ CD45RA^+^ naive (%)^$^  CD45RO^+^ memory (%)^$^ | 249 [96-450]  25  47 | **96*** [54-244]  17  49 | **342*** [302-1015]  41  43 | 650 – 1500  30 – 48  58 – 70 |
| **CD8^+^ (cells/ μl)**  CD45RA^+^CCR7^+^ naive (%)^$^  CD45RA^-^CCR7^+^ central memory (%)^$^  CD45RA^-^CCR7^-^ effector memory (%)^$^  CD45RA^+^CCR7^-^ TEMRA (%)^$^ | 353 [256-606]  13,2  3,2  54  29 | 333 [183-484]  14,6  4,3  46  36 | 380 [291-758]  10,9  1,5  68  19 | 370 – 1100  30 – 48  6 – 16  25 – 37  8 – 20 |
| **CD19^+^ (cells/ μl)** | 230 [141-405] | 187 [126-280] | 418 [180-719] | 270 - 860 |
| **CD16^+^56^+^ (cells/ μl)** | 74 [45-144] | 54 [29-145] | 90 [52-142] | 100 - 480 |
| **Immunoglobulin level,** median [IQR]  IgG (g/L)  IgA (g/L)  IgM (g/L) | 27  8 [6.7-11.1]  1.3 [0.9-1.6]  0.56 [0.3-0.8] | 15  **7.5*** [6.0-8.6]  **1.0*** [0.8-1.4]  **0.35*** [0.3-0.7] | 12  **11*** [7.3-13]  **1.5*** [1.3-2.1]  **0.7*** [0.5-1.3] | 5,5-11,5  0,4-1,6  0,5-1,5 |

*** p** < 0.05, Ig : immunoglobulin

^$^ Complete immunophenotyping available for only 6 patients (4 pre-pandemic and 2 during the pandemic)

**S5 – Initial liver and explanted liver histological features**

|  |  | Ishak score | | | | | Metavir score | | Kakuda score | | plasma cells (CD138$) | B-lymphocytes (CD20$) | | | L-lymphocytes | | | | inflammation* | hemophagocytosis | duct reaction | centra/  portal venous endothelitis |
| --- | --- | --- | --- | --- | --- | --- | --- | --- | --- | --- | --- | --- | --- | --- | --- | --- | --- | --- | --- | --- | --- | --- |
| P |  | A | B | C | D | F | A | F | bile duct loss | cholangitis activity |  | intensity | topography | organization | CD3$ | topography CD3 | % (topography) CD4 | % CD8 |  |  |  |  |
| 1 | liver explant | A4 | B6 | C4 | D2 | F1 | A3 | F1 | 0 | 0 | 0 | 0 | PA | S | +++ | PA L VCL | 10 (PA L) | 90 | PMN + | 1 | ++ | 1 |
| 2 | liver explant | A4 | B6 | C4 | D4 | F0 | A3 | F0 | 0 | 2 | 0 | ++ | PA | S | +++ | PA L VCL | 10 (PA L) | 90 | PMN + | 1 | ++ | 1 |
| 3 | liver explant | A4 | B6 | C4 | D3 | F0 | A3 | F0 | 0 | 2 | 0 | 0 | PA | S | +++ | PA L VCL | 10 (PA L) | 90 | PMN++ | 1 | +++ | 1 |
| 4 | liver explant | A4 | B6 | C4 | D4 | F0 | A3 | F0 | 0 | 1 | 0 | ++ | PA | S | +++ | PA L VCL | 10 (PA L) | 90 | PMN++ | 1 | +++ | 1 |
| 5 | liver biopsy | A4 | B4 | C4 | D3 | F2 | A3 | F1 | 0 | 2 | 0 | 0 | PA | S | ++ | PA L VCL | 10 (PA) | 90 | PMN+++ | 1 | ++ | 1 |
| 5 | liver explant | A4 | B6 | C4 | D3 | F2 | A3 | F1 | 0 | 2 | 0 | 0 | 0 | 0 | ++ | PA L VCL | 10 (PA L) | 90 | PMN+ | 1 | +++ | 1 |
| 6 | liver biopsy | NA | B6 | C4 | NA | NA | A3 | NA | NA | NA | NA | NA | NA | NA | NA | NA | NA | NA | PMN + | 0 | + | NA |
| 7 | liver biopsy | A2 | B4 | C3 | D2 | F2 | A3 | F2 | 0 | 2 | + | + | PA L | S | +++ | PA L VCL | 10 (PA L) | 90 | PMN++ | 1 | + | 1 |
| 8 | liver biopsy | A4 | B3 | C4 | D4 | F2 | A3 | F2 | 0 | 2 | 0 | ++ | PA L | S | +++ | PA L VCL | 30 (PA + L) | 70 | PMN++ | 1 | ++ | 1 |
| 9 | liver biopsy | A4 | B3 | C4 | D3 | F3 | A3 | F2 | 0 | 1 | 0 | + | PA | S | +++ | PA L VCL | 10 (PA) | 90 | PMN+++ | 1 | ++ | 1 |
| 10 | liver biopsy | A2 | B1 | C4 | D2 | F2 | A3 | F1 | 0 | 1 | NA | NA | NA | NA | NA | NA | NA |  | PMN+++ | 0 | ++ | 1 |
| 11 | liver biopsy | A4 | B6 | C4 | D3 | F3 | A3 | F2 | 0 | 2 | 0 | + | PA | S | ++ | PA L VCL | 10 (PA) | 90 | PMN+++ | 1 | ++ | 1 |
| 12 | liver biopsy | A4 | B3 | C4 | D4 | F2 | A3 | F1 | 0 | 2 | NA | + | PA | S | ++ | PA L VCL | 10 (PA) | 90 | PMN+++ | 1 | ++ | 1 |
| 13 | liver biopsy | A4 | B3 | C4 | D4 | F3 | A3 | F3 | 0 | 1 | NA | + | PA | S | ++ | PA L VCL | 20 (PA) | 80 | PMN+++ | 1 | ++ | 1 |
| 14 | liver biopsy | A3 | B3 | C3 | D3 | F2 | A3 | F2 | 0 | 2 | + | + | PA | S | ++ | PA L VCL | 10 (PA L) | 90 | PMN++ | 0 | ++ | 1 |
| 15 | liver biopsy | A4 | B4 | C4 | D4 | F2 | A3 | F2 | 0 | 2 | NA | NA | NA | NA | NA | NA | NA | NA | PMN++ | 1 | ++ | 0 |
| 16 | liver biopsy | A2 | B2 | C1 | D2 | F3 | A2 | F3 | 0 | 0 | 0 | + | PA | S | ++ | PA L VCL | 10 (PA L) | 90 | PMN+ | NA | + | 0 |
| 17 | liver biopsy | A4 | B5 | C4 | D4 | F2 | A3 | F2 | 0 | 2 | + | + | PA | S | +++ | PA L VCL | 10 (PA) | 90 | PMN +++ | 1 | ++ | 1 |
| 17 | liver explant | A4 | B6 | C4 | D3 | F2 | A3 | F2 | 0 | 2 | + | + | PA | S | ++ | PA L VCL | 10 (PA L) | 90 | PMN+ | 1 | + | 1 |
| 18 | liver biopsy | A4 | B4 | C4 | D4 | F1 | A3 | F1 | 0 | 2 | 0 | + | PA | S | +++ | PA L VCL | 20 (PA L) | 80 | PMN +++ | 1 | ++ | 1 |
| 18 | liver explant | A4 | B5 | C4 | D3 | F2 | A3 | F1 | 0 | 0 | 0 | + | PA | S | ++ | PA L VCL | 20 (PA L) | 80 | PMN+ | 0 | + | 1 |
| 19 | liver biopsy | A4 | B5 | C3 | D3 | F1 | A3 | F1 | 0 | 1 | 0 | + | PA | S | ++ | PA L VCL | 10 (PA) | 90 | PMN ++ | 0 | + | 0 |
| 19 | liver explant | NA | B6 | C4 | D2 | F2 | A3 | F1 | 0 | 1 | 0 | + | PA | S | ++ | PA L | 30 (PA L) | 70 | PMN+ | 1 | +++ | 0 |
| 20 | liver biopsy | A4 | B5 | C4 | D3 | F1 | A3 | F1 | 1 | 2 | + | + | PA L | S | +++ | PA L VCL | 20 (PA L) | 80 | PMN+ | 1 | ++ | NA |
| 21 | liver biopsy | A4 | B4 | C4 | D4 | F2 | A3 | F2 | 0 | 2 | 0 | + | PA L | S | +++ | PA L VCL | 10 (PA L) | 90 | PMN++ | 1 | +++ | 1 |
| 22 | liver biopsy | A3 | B4 | C4 | D3 | F1 | A3 | F1 | 1 | 2 | + | + | PA L | S | +++ | PA L VCL | 10 (PA L) | 90 | PMN++ | 1 | ++ | 1 |
| 23 | liver biopsy | A3 | B2 | C4 | D3 | F3 | A3 | F2 | 0 | 1 | ++ | ++ | PA L CLV | S | ++ | PA L VCL | 10 (PA) | 90 | PMN +++ | 1 | + | 1 |
| 24 | liver biopsy | A4 | B4 | C4 | D4 | F3 | A3 | F2 | 0 | 2 | + | + | PA | S | ++ | PA L VCL | 10 (PA) | 90 | PMN +++ | 1 | ++ | 1 |
| 25 | liver biopsy | A2 | B3 | C4 | D3 | F2 | A2 | F1 | 0 | 0 | + | + | PA | S | ++ | PA L VCL | 10 (PA) | 90 | PMN ++ | 1 | + | 1 |
| 26 | liver biopsy | A3 | B5 | C4 | D3 | F4 | A3 | F3 | 0 | 0 | + | + | PA | S | +++ | PA L VCL | 10 (PA) | 90 | PMN +++ | 0 | ++ | 1 |
| 27 | liver biopsy | A4 | B6 | C4 | D3 | F1 | A3 | F1 | 0 | 1 | 0 | + | PA | S | ++ | PA L VCL | 10 (PA) | 90 | PMN +++ | 1 | + | 1 |
| 28 | liver biopsy | A4 | B5 | C4 | D4 | F2 | A3 | F1 | 0 | 2 | + | + | PA | S | +++ | PA L VCL | 10 (PA L) | 90 | PMN+++ | 0 | ++ | 1 |
| 29 | liver biopsy | A4 | B4 | C4 | D4 | F3 | A3 | F2 | 0 | 2 | 0 | + | PA | S | +++ | PA L VCL | 10 (PA L) | 90 | PMN +++ | 1 | ++ | 1 |
| 30 | liver biopsy | A3 | B3 | C4 | D3 | F2 | A3 | F1 | 0 | 0 | 0 | + | PA | S | ++ | PA L VCL | 10 (PA) | 90 | PMN +++ | 0 | + | 1 |
| 31 | liver biopsy | A3 | B3 | C4 | D3 | F2 | A3 | F1 | 0 | 1 | 0 | 0 | PA | S | + | PA L VCL | 30 (PA) | 70 | PMN+++ | 0 | + | 1 |
| 32 | liver biopsy | A2 | B1 | C2 | D2 | F2 | A2 | F2 | 1 | 2 | ++ | + | PA L | S | ++ | PA L VCL | 20 (PA L) | 80 | PMN++ | 1 | ++ | 1 |

Abbreviations:

NA: not available

*other than lymphocytes

^$^ Immuno-histochemical intensity: 0 : none or rare, + : few (< 5 cells) , ++ : moderate (5-10 cells), +++ intense (> 10 cells),

F: fibrosis, P: patient, PA: portal areas, L: lobular, CLV : central lobular vein, S : scattered, PMN : Polymorphonuclear neutrophils
